# Warming-Induced Labile Carbon Change Soil Organic Carbon Mineralization and Microbial Abundance in a Northern Peatland

**DOI:** 10.3390/microorganisms10071329

**Published:** 2022-06-30

**Authors:** Lei Jiang, Xiuyan Ma, Yanyu Song, Siqi Gao, Jiusheng Ren, Hao Zhang, Xianwei Wang

**Affiliations:** 1 Key Laboratory of Wetland Ecology and Environment, Northeast Institute of Geography and Agroecology, Chinese Academy of Sciences, Changchun 130102, China; jianglei@iga.ac.cn (L.J.); maxiuyan@iga.ac.cn (X.M.); gaosiqi@iga.ac.cn (S.G.); renjiusheng256@ecit.cn (J.R.); zhanghao@iga.ac.cn (H.Z.); wangxianwei@iga.ac.cn (X.W.); 2 Xuzhou Municipal Bureau Statistics, Xuzhou 221018, China; 3 University of Chinese Academy of Sciences, Beijing 100049, China

**Keywords:** permafrost peatlands, microbial abundance, labile carbon, carbon decomposition, temperature sensitivity

## Abstract

Climate warming affects the carbon cycle of northern peatlands through temperature rises and a changing carbon availability. To clarify the effects of elevated temperature and labile carbon addition on SOC mineralization, as well as their microbial driving mechanisms, topsoil (0–10 cm) and subsoil (10–20 cm) were collected from a peatland in the Great Hing’an Mountains and incubated with or without ^13^C-glucose at 10 °C and 15 °C for 42 days. The results showed that 5 °C warming significantly stimulated SOC mineralization along with NH_4_^+^-N and NO_3_^−^-N content increases, as well as a decrease in invertase and urease activities. Glucose addition triggered a positive priming effect (PE) in the early stage of the incubation but changed to a negative PE in the late stage of the incubation. Glucose likely regulates carbon dynamics by altering fungi: bacteria, soil invertase, and β-glucodase activities, and MBC, DOC, NH_4_^+^-N contents. Glucose addition increased fungal abundance in 0–10 cm at 10 °C and 15 °C, and 10–20 cm at 10 °C, respectively, but significantly decreased fungal abundance in 10–20 cm at 15 °C. Glucose addition decreased bacterial abundance in 0–10 cm at 10 °C but increased bacterial abundance in 10–20 cm soil at 10 °C, and in 0–10 and 10–20 cm soils at 15 °C, respectively. Glucose addition significantly decreased the fungi: bacteria ratio in 0–20 cm soils at 15 °C. In addition, *Q*_10_ was significantly positively correlated with the changes in soil DOC, NH_4_^+^-N contents, invertase, and β-glucosidase activities, while negatively correlated with fungi: bacteria and urease activities after 5 °C of warming, and glucose addition significantly increased the *Q*_10_. Labile carbon may decrease carbon losses in northern peatlands that inhibit warming-induced carbon emission increase, thus partially buffering soil carbon content against change.

## 1. Introduction

Northern peatlands are characterized by cold and wet conditions that promote the accumulation of soil organic carbon (SOC) [1,2]. They contain nearly one-third of the global carbon (C) pool [3], with potential C stock reaching 875 ± 125 Pg carbon before the end of the present interglacial [4]. Due to this great C pool reserve, a slight change in the SOC mineralization of northern peatlands may have a major impact on the global carbon cycle. Moreover, SOC in northern peatlands is sensitive to climate warming [5]. Climate warming can not only stimulate SOC mineralization by affecting activities of soil microbes, enzymes, and animals [6,7,8], but also further increase plant litter, plant biomass, and root biomass into the soil [9]. The increases in the SOC activate soil microorganisms and release extracellular enzymes, resulting in large carbon emissions of native soils (priming effect, PE) or retard it (negative PE) [10,11]. A previous study reported that warming-induced increases in soil C and nitrogen (N) can enhance PE, thus reducing potential carbon sequestration [12]. Therefore, it is of great significance to study the response of SOC mineralization to interaction effects of warming in northern peatlands for evaluating the global C and N cycle under climate warming.

Root exudates play a vital role in microbially driving soil organic matter (SOM) turnover and ecosystem C cycling by providing a labile C source for microorganisms [13,14]. Glucose, oxalic acid, citric acid, and glutamic acid are the most abundant among low molecular weight root exudates [15]. Of which, glucose is the most plentiful plant sugar [16,17] and a vital carbon resource for microorganisms [18], which can shape bacterial communities and activities in the rhizosphere soil [19]. So, it is very important to clarify the mechanisms of how labile carbon mediate soil biogeochemical processes for predicting soil C stocks under global change [13].

The PE of SOC mineralization induced by exogenous carbon is an important ecological process in regulating the soil carbon cycles [20]. Previous explanations of the PE included preferential substrate utilization [21], microbial community shifts [22], nitrogen mining [23], microbial activation [24], and carbon starvation [25] mechanisms. However, so far, the most extensive explanation for the PE is that the addition of labile carbon stimulates the growth and metabolic contribution of microbial *r*-strategists, followed by the gradual increase in the abundance of *K*-strategists whose metabolic performance causes the soil organic matter decomposition [26]. Generally, the pioneering *r*-strategists are dominated by bacterial decomposers, while the effective *K*-strategist decomposers of soil organic matter are more dominated by fungi [27,28]. Changes in the direction and magnitude of PE are closely related to the changes in soil nutrient availability (especially N availability), which might influence the PE through changing the microbial biomass and enzyme activity [6,29]. 

Under climate change, the soil microbial community in peatland allocates additional labile C resources to enzyme productions to meet nutrient demands, and as such, dampens C loss through respiration [30,31]. The temperature sensitivity of CO_2_ emissions (*Q*_10_) is important for predicting changes in SOC mineralization due to warming [32]. Moreover, substrate availability and soil microbes are key to the response of *Q*_10_ to experimental warming [33,34]. Additionally, invertase, β-glucosidase, and urease are important extracellular enzymes related to the soil C and N cycles. Their activities control the decomposition of organic matter and reflect the metabolic requirements of the soil microbial community and the status of the available carbon resources [35,36]. Consequently, studying the changes in soil active carbon, available nitrogen contents, microbial and enzyme activities by warming and exogenous carbon input, as well as studying the relationship between these changes and PE or *Q*_10_, is helpful to understand the mechanism of temperature and labile carbon input effects on PE and *Q*_10_ of SOC mineralization.

In this study, we conducted a warming simulation experiment and added the ^13^C-labeled glucose to the peatland soil from the Great Hing’an Mountains, which is a typical northern permafrost region in Northeast China. The aim of our study was to investigate how labile carbon inputs affect native SOC mineralization and its temperature sensitivity, as well as their driving mechanisms in this northern peatland. We particularly examined whether microbial abundance and enzyme activity was more important in regulating the mineralization of SOC. We hypothesized that (i) the addition of glucose would induce positive PE and this effect can be stimulated by warming, and (ii) the addition of glucose would increase the *Q*_10_ values of SOC mineralization, by changing the interactions among microbial enzyme and substrates [37,38].

## 2. Materials and Methods

### 2.1. Study Area and Soil Sampling

The study area (52.94° N, 122.86° E) is located in a permafrost peatland of the Great Hing’an Mountains, Northeast China, and occurs in sloped and wide valley. It is classified as poor fen and soil type is Glacic Historthels [39]. This area belongs to the cold temperate monsoon climate zone, the average annual air temperature is −3.9 °C, average annual rainfall is 450 mm (July and August accounts for 45% of the rain falling) [40], and the altitude is 467 m. Soil covered with *Sphagnum* on surface and the vegetation is dominated by *Ledum palustre L.*, *Vaccinium uliginosum L.*, *Eriophorum vaginatum L.*, and *Sphagnum* spp. Water table in this area is relatively stable, water and soil in the permafrost active layer usually remain frozen from October to April of the next year and begin to thaw in late April [41].

In September 2016, soil samples were collected from 0–10 and 10–20 cm and were transferred immediately to the laboratory. During sampling, the soil was moist, with high moisture content but not flooded. In the laboratory, visible roots, litter, residue, stones, and gravel > 2 mm in diameter were removed and the soil samples were screened by 4 mm sieve, then initial properties were analyzed, and the remaining soils were stored at 4 °C a week prior to the incubation experiment. Physicochemical properties of the initial soils were shown in Table 1.

### 2.2. Laboratory Incubation and CO_2_ Analysis

Four treatments of different incubation temperatures and glucose additions were designed for 0–10 and 10–20 cm depth soil, respectively: 10 °C without glucose; 10 °C with ^13^C-labeled glucose; 15 °C without glucose; and 15 °C with ^13^C-labeled glucose (10 °C and 15 °C were chosen because they were close to the average temperature of the growing season across the study area). Soil samples were incubated in a 500 mL wide-mouth bottle that covered with polyethylene film, which was punctured with needle holes to maintain aerobic conditions. Each treatment contained fresh soil sample (equivalent to 10 g of oven dried soil) and repeated 4 times. Forty jars in total were prepared for the incubation experiment. Preincubation were conducted for 3 days before the addition of glucose for minimizing the influences of abrupt change in temperature on microbial composition and activity. For glucose addition treatment, 98 atom% ^13^C-glucose was diluted with unlabeled glucose to reach 25 atom% ^13^C-glucose. The root exudation rates range from 200 mg to 3000 mg per kg root per week [42,43]. The diluted glucose was added to the soil as a solution in glucose addition treatment at a rate of 1000 mg glucose kg^−1^ dry soil (equal to approximately 400 mg C kg^−1^ dry soil, approximately 30% of soil microbial biomass carbon, MBC). The same amount of deionized water was added to the control treatments. Distilled water was used to supply volatile water every week. The experiment lasted for 42 days in darkness. Soil CO_2_ was determined in the 1st, 4th, 7th, 14th, 21st, 28th, 35th, and 42nd days of the incubation. For gas sampling, the wide-mouth bottle was first opened for 20 min to let fresh air in. Then, the wide-mouth bottle was sealed with a rubber stopper with three-way valve and allowed to accumulate CO_2_ for 3 h. The headspace gas from each wide-mouth bottle was extracted by a 50 mL syringe with a 3-way value. After gas sampling, the wide-mouth bottle was left open to replenish with fresh air. The extracted gases were analyzed for their CO_2_ content by gas chromatography (Agilent 7820, Santa Clara, CA, USA) within 12 h after sampling. The isotopic signature (*δ*^13^*C*) of the CO_2_ was measured on a stable isotope mass spectrometer (Delta V Advantage, Thermo, Germany). Soil DOC (dissolved organic carbon), MBC, NH_4_^+^-N, NO_3_^−^-N contents, invertase, β-glucosidase, urease activities, bacterial and fungal abundances were analyzed at the end of the 42 days incubation.

### 2.3. Fungal and Bacterial Abundance Analysis

The bacterial and fungal abundances were determined by real-time PCR. First, the DNA from 0.3 g of soil samples was extracted from soil sample with a FastDNA spin kit (MPbio, Santa Ana, CA, USA) following the instructions of the manufacturer. Then, DNA extracts were purified by 0.5% low-melting-point agarose gels and extracted with phenol–chloroform–butanol. An ABI StepOne instrument (Applied Biosystems, San Francisco, CA, USA) using SYBR green detection chemistry was used for the real-time PCR. RT-PCR analysis for each soil sample was replicated three times. The abundance of bacteria was estimated using the primers 338F (5′-CCTACGGGAGGCAGCAG-3′) and 518R (ATTACCGC GGCTGCTGG). The abundance of fungi was estimated using the primers ITS1F (5′-CTTGGTCATTTAGAGGAAGTAA-3′) and ITS4 (5′-CAGGAGACTTGTAC ACGGTCCAG-3′). For real-time PCR, each 25 µL reaction mixture contained (i) 12.5 µL of SYBR Buffer (TaKaRa, Beijing, China); (ii) 0.4 µL of each primer (10 μM); (iii) 0.5 µL of ROXII (TaKaRa); (iv) 0.875 µL 3% BSA; (v) 0.625 µL of DMSO, and (vi) 10 ng of template DNA [44]. For producing a standard curve, the amplicon products of phylogenetic and functional markers were purified by a cyclic purification kit (Omega Bio-Tek, Norcross, GA, USA), ligated to the vector pMD18-T (TaKaRa), and subsequently transformed into *Escherichia coli*. TOP10 competent cells. The plasmid mini kit (Omega Bio-Tek) was used to extract the plasmids and using a basic local alignment search tool to detect the plasmids specificity [45]. The standard curves were generated by serial dilution of the plasmids.

### 2.4. Enzyme Activity Analysis

Soil invertase activity (mg glucose g^−1^ soil 24 h^−1^) was determined by incubating the soil for 24 h with 15 mL of 8% sucrose solution and 5 mL of phosphate buffer (pH 5.5) at 37 °C. Soil β-glucosidase activity (mg *p*NP kg^−1^ h^−1^) was determined by incubating the samples at 37 °C for 1 h with *p*-nitrophenyl-β-D-glucopyranoside (0.5 mol L^−1^) as substrate. The reaction was terminated by adding tris-(hydroxymethyl)-aminomethane [46]. Soil urease activity (mg N-NH_4_^+^ g^−1^ soil 24 h^−1^) was determined by indol blue colorimetry [47].

### 2.5. Soil MBC, DOC, NH_4_^+^-N, and NO_3_^−^-N Contents Analysis

Soil MBC was determined by chloroform fumigation [48]. Soil samples were incubated with CHCl_3_ for 24 h and extracted with K_2_SO_4_ (0.5 mol L^−1^) solution for 30 min. After filtration, carbon concentration in the extract was measured by Multi N/C 2100 TOC analyzer (Analytik Jena, Jena, Germany). MBC was calculated using the formula: MBC = *E_C_*/0.45, where *E_C_* is the difference in organic carbon content between non-fumigated and fumigated sample extracts (mg kg^−1^). DOC content was determined according to the method described by Ghani et al. [49]. The fresh soil samples were weighted in a centrifuge tube and shaken with deionized water for 30 min. High-speed centrifugation at 8000 rpm was conducted for 20 min, and the supernatant was passed through a 0.45 μm filter membrane. DOC content of the filtrate was measured using the Multi N/C 2100 TOC analyzer. Soil NH_4_^+^-N and NO_3_^−^-N were extracted using 2 mol L^−1^ KCl solution and were measured by the AA3 continuous flow chemical analyzer (Seal Analytical, Norderstedt, Germany).

### 2.6. Total Carbon (TC) and Total Nitrogen (TN) Contents Analysis

Part of soil samples were air-dried in the shade, milled, and sieved by 0.25 mm for TC and TN determination. Soil TC contents were analyzed by the Multi N/C 2100 analyzer (Analytik Jena AG, Germany). TN contents were measured by AA3 continuous flow analyzer (Seal Analytical, Germany) after wet digestion by sulfuric acid.

### 2.7. Calculations and Statistical Analysis

The SOC mineralization rate was calculated as follows [29,50],

(1) $$F=\alpha \times \frac{M}{V_{0}}\times \frac{273}{273+T}\times \frac{\Delta C}{\Delta t}\times \frac{V}{m}$$

where, *F* is the SOC mineralization rate (mg C kg^−1^ dry soil d^−1^); *α* is the coefficient of CO_2_ concentration converted to standard unit; *M* is the molar mass of CO_2_ in 1 mol CO_2_ (12 g); *V*_0_ is the molar volume (22.4 L mol^−1^) of the gas under the standard condition (temperature is 273 K, air pressure is 101 kPa); *T* is the experimental temperature (°C); Δ*C* is the change rate of CO_2_ concentration in unit time (μmol mol^−1^); Δ*t* is the airtight culture time (3 h) after air is introduced; *m* is the weight of the soil sample equivalent to dry soil (10 g); *V* is the volume of air at the top of the jar.

The cumulative SOC mineralization amount (42 days) was calculated using the following equation [29,50]:

(2) $$C_{\mathrm{soc}}=\overset{n}{\underset{i=1}{a}}\left(F_{i}A-D_{i}\right)$$

where *C*_SOC_ is the cumulative SOC mineralization; *F_i_* is the mean CO_2_ emission (carbon mineralization) rates (mg C kg^−1^ dry soil d^−1^) of the two successive sampling dates; *D*_i_ is the number of days in the sampling interval; and *n* is the number of sampling times.

The *Q*_10_ of SOC mineralization was calculated using this equation [51]:

(3) $$Q_{10}={\left(\frac{F_{i_{b}}}{F_{i_{a}}}\right)}^{\frac{10}{T_{b}a^{\wedge^{,}}Ta}}$$

where *Fi_a_* and *Fi_b_* are SOC mineralization rates at a lower temperature (*T*_a_) and a higher temperature (*T_b_*), respectively.

Cumulative SOC mineralization of native soils was calculated using the following equation [52]:

(4) $$C_{\mathrm{native}}=C_{\mathrm{treatment}}\times \left(\frac{\delta^{13}C_{\mathrm{treatment}}-\delta^{13}C_{\mathrm{glucose}}}{\delta^{13}C_{\mathrm{control}}-\delta^{13}C_{\mathrm{glucose}}}\right)$$

where *C*_native_ is the cumulative SOC mineralization derived from native soils; *C*_treatment_ is the cumulative SOC mineralization of total soil (both native soil and the added glucose); *δ*^13^*C*_treatment_ is *δ*^13^*C* values of the total soil derived C-CO_2_; *δ*^13^*C*_glucose_ is *δ*^13^*C* values of the added glucose derived C-CO_2_; and *δ*^13^*C*_control_ is *δ*^13^*C* values of SOC derived C-CO_2_ from control soil without glucose.

The PE of native SOC mineralization was evaluated as follows [53]:

(5) $$\mathrm{PE}=100\times \frac{C_{\mathrm{native}}-C_{\mathrm{control}}}{C_{\mathrm{control}}}$$

where *C*_native_ is the cumulative SOC mineralization derived from native soils with glucose treatment; and *C*_control_ is the cumulative SOC mineralization derived from native soils without glucose treatment.

### 2.8. Statistical Analyses

Figures were drawn by Origin 2017 (OriginLab, Northampton, MA, USA). Statistical analyses were performed using SPSS 20.0 (International Business Machines Corporation, Armonk, NY, USA). One-way ANOVAs were performed to examine the effects of temperature or glucose on soil active carbon, available nitrogen contents, enzyme activities, and microbial abundances. The relationship between the PE (or *Q*_10_) and changes in active carbon, available nitrogen contents, enzyme activities, and microbial abundances were examined by linear regressions. All data were checked to be normally distributed before the ANOVA and linear regression (Shapiro–Wilk test). Spearman’s correlation analysis was conducted to calculate the relationships among total cumulative native SOC mineralization amounts, the changes in soil active carbon, available nitrogen contents, and enzyme activities after incubation.

## 3. Results

### 3.1. The Effects of Warming and Glucose Addition on SOC Mineralization

**Figure 1:**
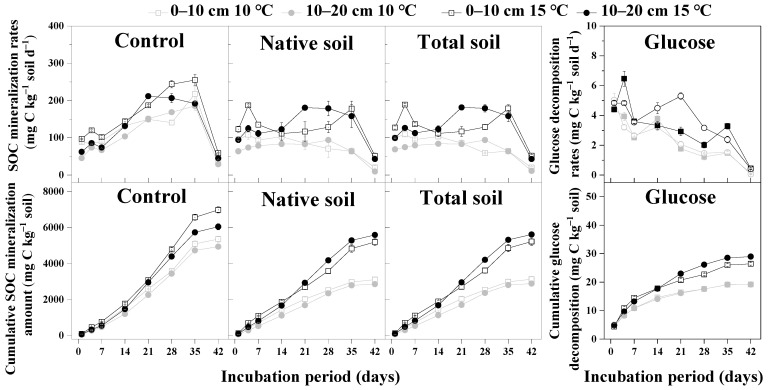
Rates and cumulative amounts of soil organic carbon mineralization and glucose decomposition (mean ± SE, *n* = 4). Control indicates soil without glucose addition treatment; native soil indicates original soil under glucose addition treatment; total soil indicates both native soil and added glucose.

Warming by 5 °C increased the SOC mineralization rates and the cumulative amount during 42 days of incubation in 0–10 and 10–20 cm soil layers both with or without glucose addition treatment (Figure 1). Total cumulative native SOC mineralization amount of 42 days incubation in 0–10 and 10–20 cm soil layers at 15 °C, respectively, increased by 1623.96 and 1112.31 mg C kg^−1^ at 10 °C, and increased 2084.31 and 2732.01 mg C kg^−1^, respectively, under glucose (Figure 1).

At 10 °C, the total cumulative native SOC mineralization amount in the 0–10 and 10–20 cm soil with glucose is, respectively, 2247.50 and 2079.35 mg C kg^−1^ lower than the control. At 15 °C, the total SOC mineralization amount in the 0–10 and 10–20 cm soil with glucose is, respectively, 1787.14 and 459.46 mg C kg^−1^ lower than the control (Figure 1). Therefore, glucose addition decreased the total cumulative native SOC mineralization amount.

### 3.2. Priming Effect

**Figure 2:**
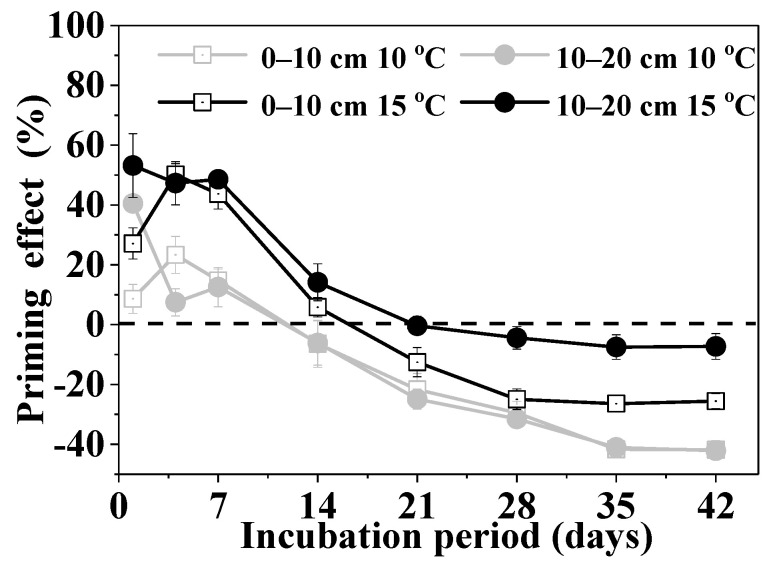
Priming effect of soil organic carbon mineralization at different temperatures and depths during 42 days incubation (mean ± SE, *n* = 4).

**Figure 3:**
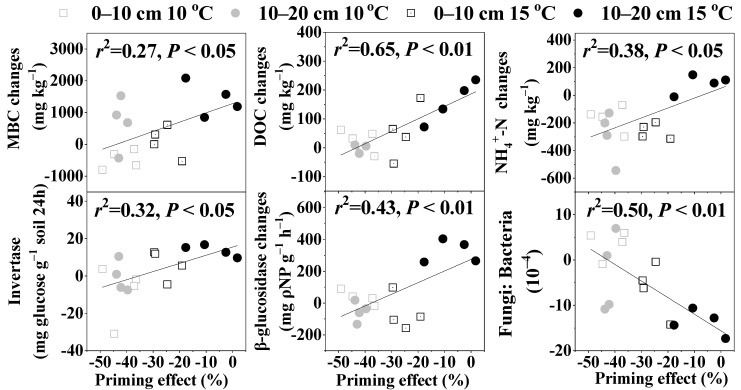
Relationships between the priming effect and the changes in soil fungi: bacteria abundance ratio, invertase and β-glucosidase activities, as well as MBC (microbial biomass carbon), DOC (dissolved organic carbon), and NH_4_^+^-N contents after glucose addition.

Glucose addition induced a positive PE on SOC mineralization at the early stage of incubation (0–14 days), and 5 °C warming promoted the positive PE. However, the effects reduced gradually and were slightly negative at the late stage of the incubation (21–42 days) and warming by 5 °C inhibited the negative PE (Figure 2). The PE at the end of the incubation was significantly positively correlated with the changes in MBC, DOC, and NH_4_^+^-N contents, invertase and β-glucosidase activities, as well as the fungi: bacterial abundance ratio after glucose addition (Figure 3).

### 3.3. Temperature Sensitivity of SOC Mineralization

**Figure 4:**
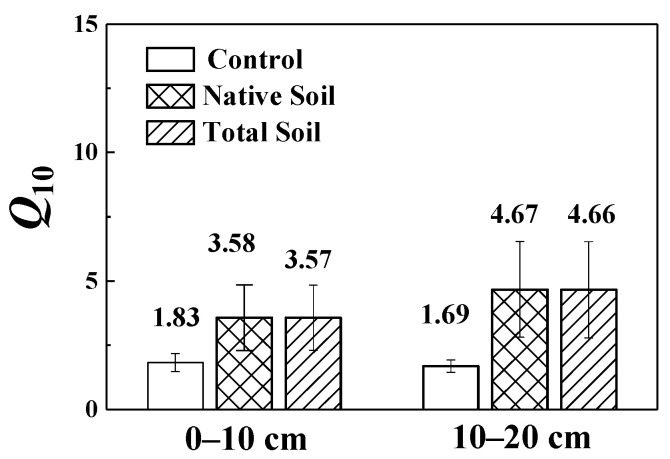
*Q*_10_ values of soil organic carbon mineralization at different depths under different treatments.

**Figure 5:**
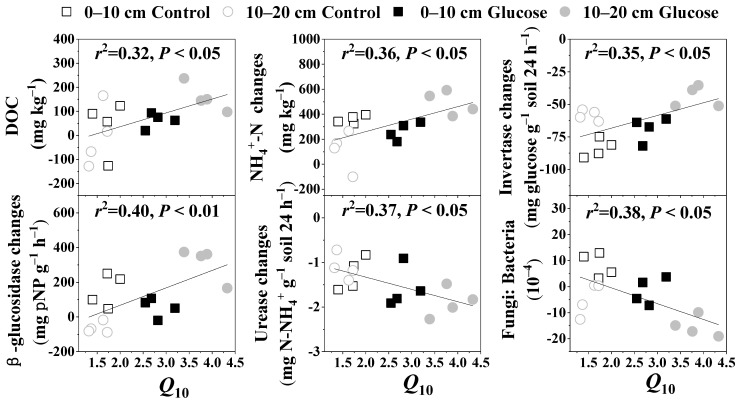
Relationships between the *Q*_10_ values and the changes in fungi: bacteria abundance ratio, enzyme activities, and DOC (dissolved organic carbon) and NH_4_^+^-N contents after 5 °C warming.

*Q*_10_ values in 0–10 cm soil layers with or without glucose addition were 3.57 (native soil is 3.58) and 1.83, respectively, while in 10–20 cm they were 4.66 (native soil is 4.67) and 1.69, respectively (Figure 4). Glucose addition increased the temperature sensitivities of SOC mineralization in both soil layers. The *Q*_10_ value was significantly positively correlated with the changes in DOC and NH_4_^+^-N contents, as well as invertase and β-glucosidase activities after 5 °C of warming, but significantly negatively correlated with the changes in urease activity and the fungi: bacteria ratio (Figure 5).

### 3.4. The Effects of Warming and Glucose Addition on Fungal and Bacterial Abundances

**Figure 6:**
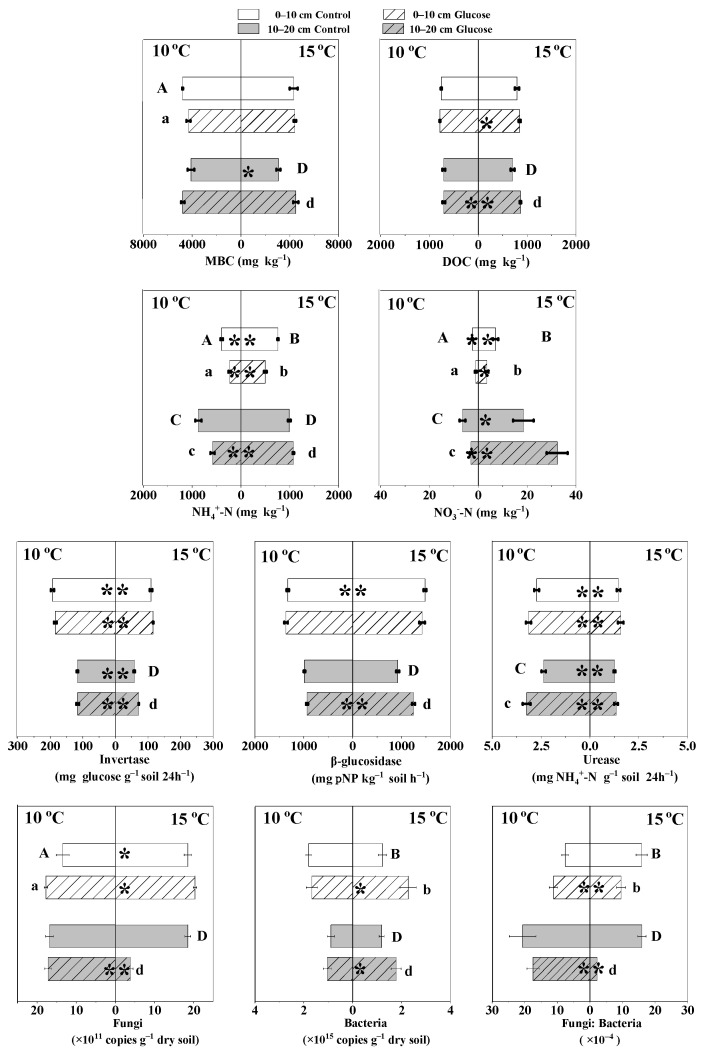
Soil microbial abundance, enzyme activity, and labile carbon, available nitrogen contents, after 42 days incubation (mean ± SE, *n* = 4). ** and *, respectively, indicate significant difference at 0.01 and 0.05 level of different temperature; same letter case indicates significant difference at 0.05 level of different glucose treatment.

Warming by 5 °C significantly increased the soil fungal abundance in 0–10 cm with or without glucose addition and increased by 10.14% in 10–20 cm soil without glucose, but significantly decreased the fungi abundance of soil in 10–20 cm with glucose (Figure 6). Warming by 5 °C decreased 32.44% of soil bacterial abundances in 0–10 cm with control but increased the bacterial abundance in both soil layers with glucose and in 10–20 cm with the control. Warming by 5 °C significantly increased the fungi: bacteria ratio in 0–10 cm without glucose addition but decreased the fungi: bacteria ratio in 0–10 cm with glucose and 10–20 cm soils. Glucose addition increased 31.60%, 9.71%, and 2.19% of fungal abundance in 0–10 cm at 10 °C and 15 °C, and 10–20 cm at 10 °C, respectively, but significantly decreased 77.68% of soil fungal abundance in 10–20 cm at 15 °C. Glucose addition decreased 7.53% of bacterial abundance in 0–10 cm at 10 °C, but increased 16.02%, 86.77%, and 49.76% of bacterial abundance in 10–20 cm soil at 10 °C, and in 0–10 and 10–20 cm soils at 15 °C, respectively. Glucose addition significantly decreased the fungi: bacteria ratio in 0–10 and 10–20 cm soils at 15 °C.

### 3.5. The Effects of Warming and Glucose Addition on MBC and DOC Contents

MBC contents in 0–10 and 10–20 cm soil layers at 15 °C, respectively, decreased 462.08 and 1030.14 mg kg^−1^ at 10 °C under the control, while increased 117.09 mg kg^−1^ in 0–10 cm and decreased 281.25 mg kg^−1^ in 10–20 cm under glucose addition (Figure 6). Warming by 5 °C had no significant effect on DOC contents with the control, but significantly increased DOC contents with glucose addition in both soil layers.

### 3.6. The Effects of Warming and Glucose Addition on NH_4_^+^-N and NO_3_^−^-N Contents

NH_4_^+^-N contents in 0–10 and 10–20 cm soil layers at 15 °C, respectively, increased 359.38 and 115.09 mg kg^−1^ at 10 °C under the control, and, respectively, increased 265.90 and 490.55 mg kg^−1^ under glucose addition (Figure 6). NO_3_^−^-N contents in both soil layers with and without glucose addition were also significantly increased by 5 °C warming. Glucose addition significantly decreased the NH_4_^+^-N and NO_3_^−^-N contents in both soil layers at 10 °C and the 0–10 cm soil layer at 15 °C, but increased NH_4_^+^-N and NO_3_^−^-N contents in the 10–20 cm soil layer at 15 °C. Changes in NH_4_^+^-N and NO_3_^—^N contents with 42 days of incubation significantly positively correlated with the total cumulative SOC mineralization amount (Table 2).

### 3.7. The Effects of Warming and Glucose Addition on Invertase, β-glucosidase, and Urease Activities

Warming by 5 °C significantly decreased invertase and urease activities whether or not we added glucose in both soil layers, but significantly increased β-glucosidase activities in the 0–10 cm soil layer without glucose, and in the 10–20 cm soil layer with glucose (Figure 6). Glucose addition significantly increased invertase and β-glucosidase activities at 15 °C in 10–20 cm, and increased urease activities at 10 °C and 15 °C in both soil layers (Figure 6). Changes in invertase and urease activities with 42 days of incubation significantly negatively correlated with the total cumulative native SOC mineralization amount, and NH_4_^+^-N and NO_3_^−^-N contents (Table 2).

## 4. Discussion

### 4.1. Response of SOC Mineralization to Warming and Its Temperature Sensitivity

The incubation temperature had a strong, positive effect on SOC mineralization both with or without glucose addition treatment, indicating that SOC mineralization in peatland of the Great Hing’an Mountains was strongly limited by temperature. Along with the increased total cumulative SOC mineralization amount, we also observed that with elevated temperature NH_4_^+^-N and NO_3_^−^-N contents increased, and invertase and urease activities decreased. Additionally, Spearman’s correlation analysis showed a significant relationship between them. This suggested that the elevated temperature promoted both the soil carbon and nitrogen mineralization in this peatland soil, and the increase in NH_4_^+^-N and NO_3_^−^-N contents may further stimulate the microbial-mediated mineralization rates of carbon, leading to the continuous consumption of soil enzyme substrates, thus reducing invertase and urease activities [54].

*Q*_10_ is a key parameter to predict the future soil carbon dynamics by temperature [55]. Previous studies have shown that the availability of soil substrate can affect the *Q*_10_ values of SOC [56,57]. In this study, glucose addition obviously increased *Q*_10_ values of SOC mineralization. As *Q*_10_ values increased with increasing SOC recalcitrance (stability) [58,59,60], we infer that increased *Q*_10_ values may be attribute to the formation and increment of recalcitrant SOC promoted by glucose, which further increases the activation energy of the soil, thus increasing the *Q*_10_ values [61]. Creamer et al. [62] also reported that exogenous carbon input increased the *Q*_10_ of SOC mineralization, and soil microbial abundances had a certain impact on carbon mineralization [63]. In our study, we also observed a significantly negative relationship between the *Q*_10_ values and the changes in fungi: bacteria abundance ratio after 5 °C warming. In addition, the *Q*_10_ value was significantly positively correlated with the changes in invertase and β-glucosidase activities after 5 °C warming in our experiment. Although we observed that the 5 °C warming significantly reduced the invertase activity, the less the invertase activity decreased, the higher the *Q*_10_ was.

Consistent with our hypothesis, we found that glucose addition increased the *Q*_10_ values of SOC mineralization by changing the interactions among the microbial enzymes and substrates. This is because the SOC mineralization is an enzymatic reaction process driven by microorganisms. The higher the enzyme activity relatively is, the more favorable the SOC mineralization and the higher the *Q*_10_. Additionally, we also observed a significantly positive correlation between the *Q*_10_ value and changes in the DOC and NH_4_^+^-N contents after 5 °C of warming. Overall, the *Q*_10_ value of SOC is affected by the common effects of the soil available carbon and nitrogen contents, the enzyme activity, and the microbial community composition.

### 4.2. Response of SOC Mineralization to Glucose Addition and Its Microbial Mechanism

Inconsistent with our first hypothesis, the response of PE to glucose addition depends on the incubation period. A positive PE of SOC mineralization was observed in glucose addition soils in the early stage of the incubation, followed by a negative effect. Such a change in the direction of the PE might be because in the early stage of the incubation, soil microorganisms associated with SOC mineralization were stimulated by the large amounts of glucose, but due to the limitation of nitrogen and other elements, microorganisms need to decompose more organic matter to obtain nitrogen and other nutrition, thus producing a positive PE [23]. A so called “nitrogen mining” mechanism [23].

However, the mechanism of the subsequent negative PE was relatively elusive. It was likely that multiple mechanisms coexist, and the mechanisms may vary with different soil layers and temperatures. It is known that the fate of glucose is usually determined by respired carbon, microbial biomass, DOC, and SOC, and that the microbial immobilization of glucose out-competes physico-chemical sorption in soil [56]. The amount of added glucose was equivalent to 400 mg C kg^−1^ dry soil in our experiment, but the glucose-derived carbon losses through CO_2_ release during incubation was only approximately 19.16–28.95 mg C kg^−1^, and the increment of DOC contents was only 160 mg kg^−1^ at most. Given that glucose can effectively incorporate into microbial biomass [64], the other part of glucose was likely to enter the body of microorganisms or be polymerized by microorganisms into recalcitrant SOC and fixed to the soil. A previous study supporting our view was that under the condition of low concentrations, glucose-derived carbon was stored in a transitional pool (metabolic arrest) and not decomposed to CO_2_ [65]. Additionally, Hopkins et al. [66] found that the added glucose may be transformed into microbial residues with various complexities in chemical structure. Moreover, Yuan et al. [67] observed that glucose addition resulted in a net C accumulation, which challenged the assumption that glucose serves as a co-metabolite in driving SOM decomposition to lose C.

It is reasonable that the pathway of glucose consumption was dominated by polymerization into recalcitrant SOC, and the negative PE of native SOC mineralization in the later stage of the incubation can be also naturally attributed to the formation of refractory SOC. Interestingly, glucose addition decreased 7.53% of the bacterial abundance, which was much greater than the increased fungal abundance of 0–10 cm soil at 10 °C. As soil microorganisms were dominated by bacteria, which can both specifically utilize exogenous labile carbon and native SOC, the reduction of bacterial abundance was also an important reason for the reduction of native SOC mineralization. The report of de Graaff et al. [27] also found that labile carbon addition can regulate the decomposition of more recalcitrant soil carbon by controlling the relative abundance and activity of fungi and bacteria. For soil in 10–20 cm at 10 °C and 0–10 cm at 15 °C, glucose addition increased MBC contents by 16.49% and 2.28%, respectively; increased bacterial abundance by 16.02% and 86.77%, respectively; and increased fungal abundance by 2.19% and 9.71%, respectively. However, glucose addition significantly decreased the available nitrogen (NH_4_^+^-N and NO_3_^—^N) contents. This demonstrated that glucose addition stimulated soil microbial abundances at these conditions, and with the growth of microorganisms as well as the consumption of glucose and soil substrate (available nitrogen), the microbial activities were gradually limited, weakening the decomposition of the native soil organic matter in soils, thus leading to the negative PE at the later stage of the incubation [68]. For the soil in 10–20 cm at 15 °C, we observed that the addition of glucose obviously increased the MBC, DOC, and available nitrogen contents, enzyme activities, and *r*-strategy microorganisms’ abundance, but decreased the *K*-strategy microorganisms’ abundance. This suggested that the root exudate carbon input may stimulate the nitrogen mineralization in 10–20 cm soil at 15 °C, and the increased available nitrogen can relatively meet the needs of microorganisms, then microorganisms reduced the mining of nitrogen through the decomposition of the native soil organic matter, thus expressing a negative PE.

Finally, regardless of the mechanisms implied, our linear regression analysis showed that the final PE had a significant positive relationship with the changes of MBC, DOC, and NH_4_^+^-N contents, and invertase and β-glucosidase activities under glucose addition, and had a significant negative relationship with the changes in the fungi: bacteria ratio, suggesting that glucose addition may control the direction and intensity of the priming effect by affecting the soil active carbon and available nitrogen contents, carbon cycle-related enzyme activities, as well as microbial community structure. This conclusion supported our above conjecture about why the PE direction switched from positive to negative. Consistent with our findings, Qiu et al. [69] found that the PE induced by the input of labile organic carbon was regulated by the soil available nitrogen and Xiao et al. [70] also found that the PE linearly correlated with the MBC response to exogenous carbon input. Besides, a previous meta-analysis demonstrated that the direction and magnitude of the PE depended on the ratio of the added substrate amount to the size of the MBC, when the added amount of available organic carbon was higher than 50% of the microbial biomass carbon, an exponential decrease in the PE or even a switch to negative values was always observed [26]. Here, the added glucose amount was equivalent to 28.49% (in 0–10 cm) and 27.39% (in 10–20 cm) of MBC, thus broadening the range of the added glucose concentration that may lead to a negative PE.

Specifically, the total cumulative SOC mineralization amount of native soils at 15 °C under glucose addition was similar to that at 10 °C without glucose, indicating that the added glucose to soil counteracted the increased total cumulative SOC minimization amount caused by 5 °C of warming. Therefore, in the long run, the priming effect may decrease native soil carbon losses in northern peatlands, which exhibit warming-induced carbon decreases, thus partially buffering soil carbon content against change. However, in the natural conditions, labile carbon is continuously released by the roots, and field research is needed to clarify the long-term response of soil carbon loss under warming.

## 5. Conclusions

Three key conclusions can be derived from the findings of this study. First, cumulative native SOC mineralization in northern peatlands increased with elevated temperature, which may be largely a result of warming-induced increase in available nitrogen supply, and this process was accompanied with soil invertase and urease activities decreasing. Second, in the long run, a glucose-induced negative PE can counteract the warming-induced carbon losses in northern peatlands, and the PE was correlated with the changes in MBC, DOC, NH_4_^+^-N contents, invertase and β-glucosidase activities, as well as fungi: bacteria by glucose input. Third, *Q*_10_ values were significantly positively correlated with the changes in soil DOC, NH_4_^+^-N contents, and invertase and β-glucosidase activities, but significantly negatively correlated with changes in urease activities and the fungi: bacteria ratio by 5 °C of warming, and glucose addition significantly increased the *Q*_10_. Therefore, the increase in labile carbon input caused by global change may increase the temperature sensitivity of SOC mineralization in northern peatlands, but in the long run, it may not increase soil carbon release to the atmosphere. This study is based on short-term indoor culture experiments. Future research should focus on the effects of temperature rise and plant root carbon input on soil microbial carbon decomposition and fixation based on long-term field observations and control experiments.

## Figures and Tables

**Table 1:** Physicochemical properties (mean ± SE, *n* = 4) of initial soils. TC: total carbon content; TN: total nitrogen content; MBC: dissolved organic carbon; DOC: dissolved organic carbon.

Depth (cm)	0–10	10–20
Water content (%)	83.50 ± 2.08	82.66 ± 2.16
TC (mg g^−1^)	366.33 ± 8.20	384.45 ± 14.57
TN (mg g^−1^)	15.65 ± 0.52	18.68 ± 1.06
MBC (mg kg^−1^)	1421.60 ± 482.45	1478.70 ± 85.86
DOC (mg kg^−1^)	620.25 ± 81.92	338.42 ± 44.03
NH_4_^+^-N (mg kg^−1^)	66.60 ± 17.05	47.91 ± 6.54
NO_3_^−^-N (mg kg^−1^)	0.55 ± 0.07	0.58 ± 0.09
Invertase (mg glucose g^−1^ soil 24 h^−1^)	324.84 ± 23.30	213.98 ± 50.37
β-Glucosidase (mg *p*NP kg^−1^ h^−1^)	2215.30 ± 41.75	2732.10 ± 47.49
Urease (mg N-NH_4_^+^ g^−1^ soil 24 h^−1^)	4.88 ± 0.57	3.21 ± 0.78

**Table 2:** Spearman’s correlation analysis among total cumulative SOC (soil organic carbon) mineralization amount of native soils, changes in MBC (microbial biomass carbon), DOC (dissolved organic carbon), NH_4_^+^-N, NO_3_-N contents, invertase, β-glucosidase, and urease activities after incubation. * indicated significant at 0.05 level, ** indicated significant at 0.01 level.

	MBC	DOC	NH_4_^+^-N	NO_3_-N	Invertase	β-Glucosidase	Urease
Cumulative SOC mineralization	−0.25	−0.02	0.46 **	0.62 **	−0.62 **	0.25	−0.62 **
MBC		−0.26	−0.35	−0.35 *	0.23	0.17	0.07
DOC			0.78 **	0.66 **	0.25	−0.58 **	0.44 *
NH_4_^+^-N				0.94 **	−0.04	−0.46 **	0.11
NO_3_-N					−0.24	−0.28	−0.12
Invertase						−0.54 **	0.84 **
β-Glucosidase							−0.72 **

## Data Availability

Data are available on request.
